# Pathogenicity and molecular characterization of a fowl adenovirus 4 isolated from chicken associated with IBH and HPS in China

**DOI:** 10.1186/s12917-018-1733-4

**Published:** 2018-12-14

**Authors:** Limin Li, Jianchang Wang, Ping Chen, Shan Zhang, Jiguo Sun, Wanzhe Yuan

**Affiliations:** 1 College of Veterinary Medicine, Hebei Agricultural University, Baoding, 071001 Hebei China; 2 Hebei Engineering and Technology Research Center of Veterinary Biotechnology, Baoding, 071001 Hebei China; 3 North China Research Center of Animal Epidemic Pathogen Biology, China Agriculture Ministry, Baoding, 071001 Hebei China; 4 Inspection and Quarantine Technical Center of Hebei Entry-Exit Inspection and Quarantine Bureau, 318 Heping West Rd, Xinhua District, Shijiazhuang, 050051 Hebei China

**Keywords:** Fowl adenovirus 4, Hydropericardium syndrome, Inclusion body hepatitis, Virulent, Pathogenicity, Phylogenetic analysis, Chickens

## Abstract

**Background:**

Since July in 2015, an emerging infectious disease, Fowl adenovirus (FAdV) species C infection with Hepatitis-Hydropericardium syndrome was prevalent in chicken flocks in China. In our study, one FAdV strain was isolated from commercial broiler chickens and was designated as SDSX1.The phylogenetic information, genetic mutations and pathogenicity of SDSX1 were evaluated.

**Results:**

The phylogenetic analysis indicated that SDSX1 is a strain of serotype 4, FAdV-C. The amino acid analysis of fiber-2 showed that there were more than 20 mutations compared with the non-virulent FAdV-C strains. The pathogenic evaluation of SDSX1 showed that the mortality of one-day-old chickens inoculated SDSX1 was 100%. The typical histopathological changes of SDSX1 were characterized by the presence of basophilic intranuclear inclusion bodies in hepatocytes. The virus copies in different tissues varied from10^7^ to 10^11^ per 100 mg tissue and liver had the highest virus genome copies.

**Conclusion:**

In conclusion, the isolate SDSX1, identified as FAdV-4, could cause one-day-old chicks’ typical inclusion body hepatitis (IBH) and hepatitis-hydropericardium syndrome (HHS) with 100% mortality. The virus genome loads were the highest in the liver. Molecular analysis indicated that substitutions in fiber-2 proteins may contribute to the pathogenicity of SDSX1.

## Background

Fowl adenoviruses (FAdVs) are non-enveloped, double-stranded DNA linear viruses, belonging to the genus *Aviadenoviru*sof the family *Adenoviridae* [1, 2]. The viral genome is approximately 43–46 kb, encoding a number of structural and nonstructural proteins [3–5]. The virion capsid consists of three major structural proteins, hexon, fiber and penton base proteins. Hexon is the major protein of the adenovirus capsid known to have a region related to virus neutralization and serotype specificity.

FAdVs are grouped into 5 species (FAdV-A to FAdV-E) based on genomic restriction enzyme digest patterns with 12 serotypes identified so far: FAdV-A (serotype FAdV-1); FAdV-B (serotype FAdV-5);FAdV-C (serotypes FAdV-4 and -10); FAdV-D (serotypes FAdV-2, − 3, − 9, and − 11); and FAdV-E (serotypes FAdV-6, − 7, −8a, and -8b) [6]. FAdVs have a worldwide distribution, but different serotypes or genotypes are discovered in different geographic regions [7–11]. FAdVs infection (the strain was closely related to serotype 11 and 8a) was first reported in Morocco recently [12]. All 12 serotypes of FAdVs have been associated with the outbreak of inclusion body hepatitis (IBH) with a ~ 10% mortality rate [7]. Chickens between 3 and 4 weeks old are especially vulnerable to IBH that is characterized by congested and enlarged liver with hepatocyte necrosis and petechial hemorrhage. As the pathognomonic lesion of FAdVs infection, basophilic intranuclear inclusion bodies are usually observed in degenerated hepatocytes surrounded by a clear halo or filling the entire nucleus [13]. FAdV-4 has been implicated in hepatitis-hydropericardium syndrome (HHS) characterized by accumulation of transparent or straw-colored fluid in the pericardial sac, nephritis, and hepatitis with a 30–100% mortality rate [14–16]. Also, pathogenicity assays confirmed that FAdV-4 could induce HHS [8].

From June to November in 2015, a disease emerged in large-scale broiler and egg-laying chickens in farms in Shandong, Henan, Hebei, Liaoning, Anhui and Jilin provinces in China. The disease inflicted mostly the 20-to-30-day-old broilers with a mortality rate of 20–30%, and occasionally affected layers aged 20–70 days or 200–300 days but with a lower mortality rate. Usually, there were no obvious clinical signs with a sudden death. The death peak was 4–8 days after onset and the course of disease could last 8–15 days. The diseased animals had flabby hearts with straw-colored fluid in the pericardial sac and the swollen liver with foci of hemorrhage and/or necrosis. The aim of this study was to molecular characterization and pathogenicity assessment of FAdV strain responsible for disease outbreak in chickens.

## Results

### Sequence analysis

**Fig. 1:**
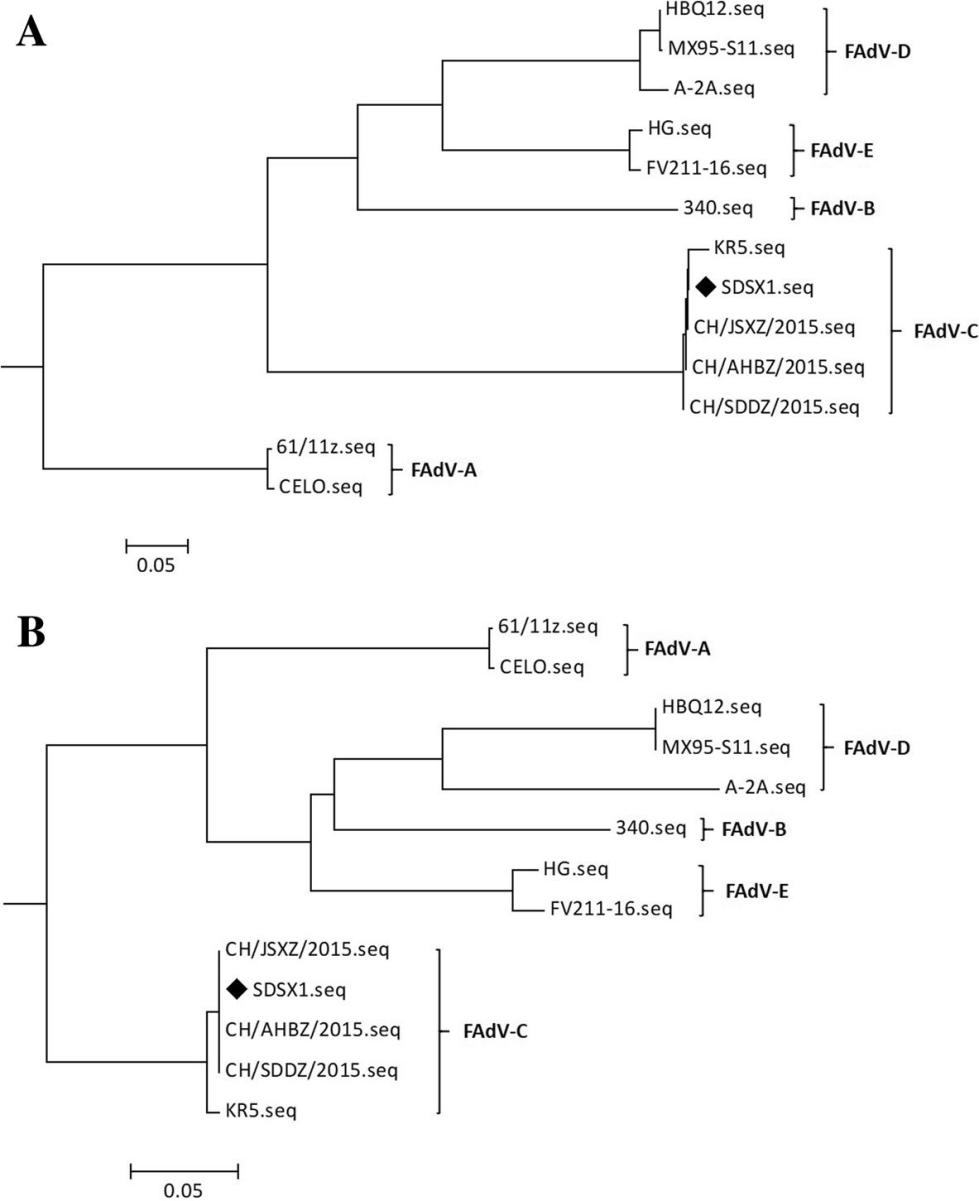
The phylogenetic analysis of the isolate SDSX1. Phylogenetic trees of SDSX1 were constructed based on the whole genome sequence (**a**) and hexon gene sequence of FAdVs strains (**b**). FAdV isolate evaluated in the study was labeled with “♦”

The virus was isolated from the SPF chicken’s embryos by 5 blind passages and it was designated as SDSX1. The whole genome nucleotide sequence and hexon gene sequence of SDSX1 are available in the GenBank (accession number is KY636400 andKT932640, respectively) and the complete genome for SDSX1 was found to be 43,630 bp in length. The percent sequence identity for available whole genomes showed that SDSX1 has 100% sequence homology with three isolates from China (CH/SDDZ/2015, CH/AHBZ/2015, and CH/JSXZ/2015, [17] and 98.6% sequence identity with a KR5 isolate from Austria. Nevertheless, SDSX1 showed a low sequence identity (< 57.0%) with the strains of other FAdV species. According to the phylogenetic analysis based on the full genome (Fig. 1a), the SDSX1 isolate was classified into serotype 4 of FAdV-C. The similar evolutionary relationships were observed from the phylogenetic tree constructed based on the hexon gene (Fig. 1b).

**Fig. 2:**
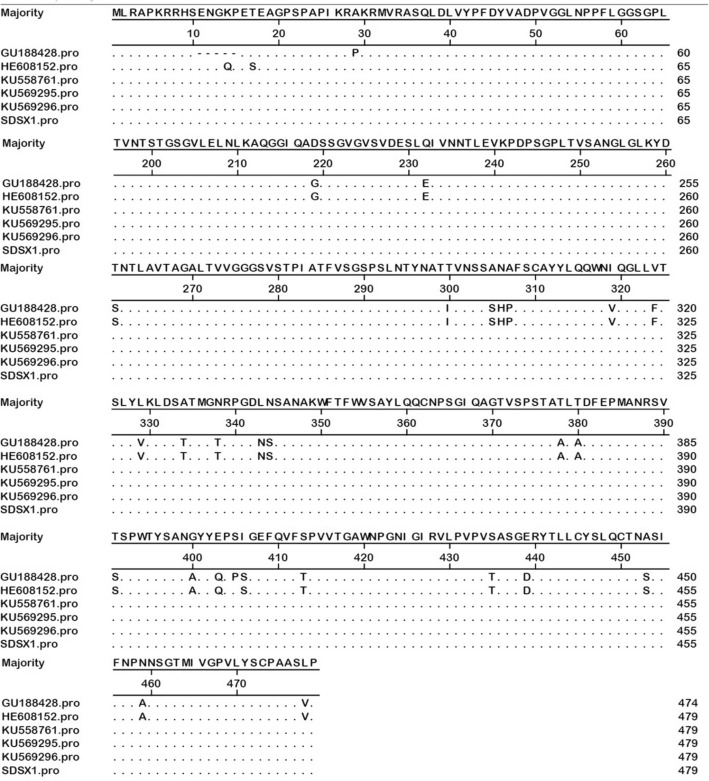
Alignment of amino acids residues of fiber-2 of SDSX1 isolate with other FAdV-C strains. Dots indicate conserved residues; dashes indicate deleted amino acids

The size of fiber-2 in SDSX1 isolate is 479 amino acid residues, the same as the strain KR5 (HE608152, FAdV-C), CH/SDDZ/2015(KU558761, FAdV-C), CH/AHBZ/2015 (KU569295, FAdV-C), and CH/JSXZ/2015(KU569296, FAdV-C) (Table 1) and 5 amino acid residues more than ON1(GU188428, FAdV-C) (Fig. 2). The basic residues rich sequence KRPK/KRAK (site 27–30) and VYPF (site 41–45) in fiber proteins of SDSX1were identified. Particularly, there were more than 20 substitutions compared with the non-pathogenic strains, ON1 and KR5 (Table 2 and Fig. 2) and shared only 94.1%~ 94.2% with ON1 and KR5, respectively. The fiber-2 protein identity of SDSX1 is 100% compared with CH/SDDZ/2015, CH/AHBZ/2015, and CH/JSXZ/2015.

**Table 1:** Reference isolates of FAdVs in the study

FAdV isolates	GenBank accession number	Geographic origin	Viral species
61/11z	KX247012	Poland	FAdV-A
CELO	U46933	Austria	FAdV-A
340	KC493646	Northern Ireland	FAdV-B
ON1	GU188428	Canada	FAdV-C
KR5	HE608152	Austria	FAdV-C
CH/SDDZ/2015	KU558761	China	FAdV-C
CH/AHBZ/2015	KU569295	China	FAdV-C
CH/JSXZ/2015	KU569296	China	FAdV-C
A-2A	AF083975	Canada	FAdV-D
HBQ12	KM096545	China	FAdV-D
MX95-S11	KU746335	Mexico	FAdV-D
HG	GU734104	Canada	FAdV-E
FV211–16	KX258422	Peru	FAdV-E

**Table 2:** Amino acid substitutions of fiber-2 in FAdV-C strains with different pathogenicity

Sites of amino acid	ON1 (GU188428)	KR5 (HE608152)	CH/SDDZ/2015 (KU558761)	CH/AHBZ/2015 (KU569295)	CH/JSXZ/2015 (KU569296)	SDSX1 (KY636400)
11–15	–	ENGQP	ENGKP	ENGKP	ENGKP	ENGKP
29	P	A	A	A	A	A
219	G	G	D	D	D	D
232	E	E	Q	Q	Q	Q
261	S	S	T	T	T	T
300	I	I	T	T	T	T
305–307	SNP	SNP	ANA	ANA	ANA	ANA
319	V	V	I	I	I	I
324	F	F	V	V	V	V
329	V	V	L	L	L	L
334	T	T	A	A	A	A
338	T	T	N	N	N	N
343–344	NS	NS	LN	LN	LN	LN
378	A	A	T	T	T	T
380	A	A	T	T	T	T
391	S	S	T	T	T	T
400	A	A	G	G	G	G
403	Q	Q	E	E	E	E
405	P	S	S	S	S	S
406	S	S	I	I	I	I
413	T	T	S	S	S	S
435	T	T	S	S	S	S
439	D	D	E	E	E	E
453	S	S	A	A	A	A
459	A	A	N	N	N	N
478	V	V	L	L	L	L

### Pathogenicity analysis of SDSX isolate

**Fig. 3:**
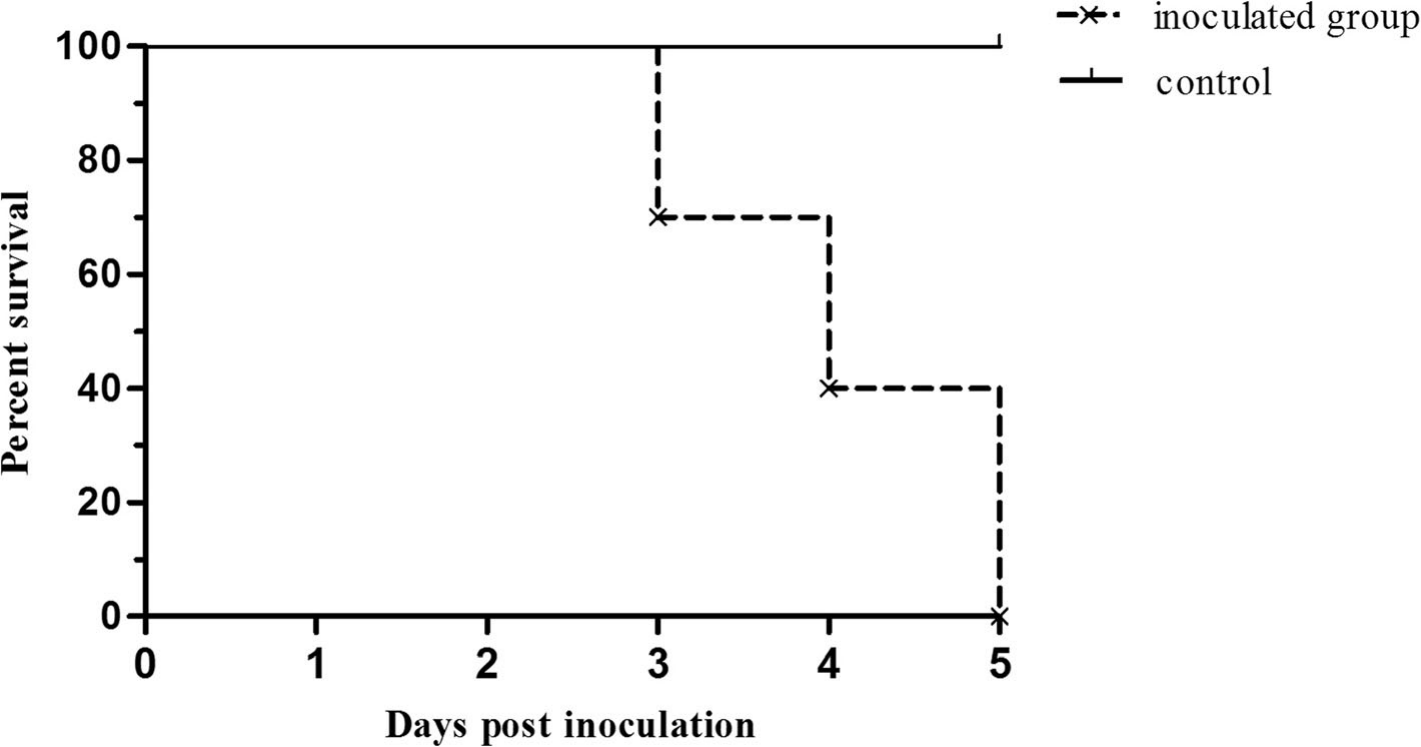
The survival curve of the inoculated and control chickens. “×” indicates the censored observation. Three, three and four chickens were censored at 3, 4 and 5 days post inoculation, respectively

**Fig. 4:**
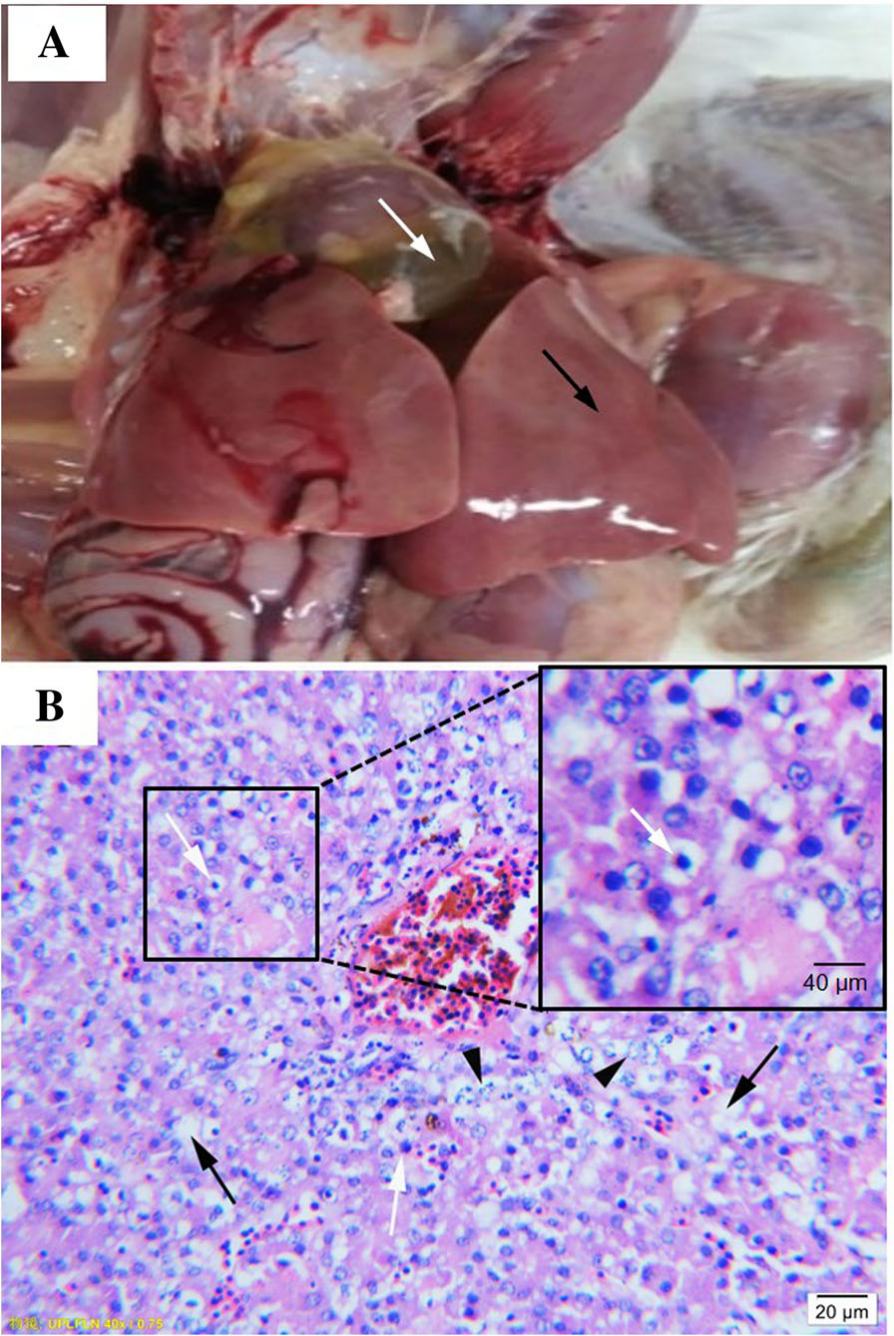
Gross pathological changes and histopathological analysis. **a** showed a representative image showing the enlarged liver with fatty degeneration (black arrow) and the heart with straw-colored fluid in the pericardial sac (white arrow); **b** showed the histopathological analysis (HE staining) of the liver tissue. Hepatocyte fatty degeneration (black arrow); basophilic inclusion bodies in the hepatocyte (white arrow); hepatocyte necrosis (arrowhead) were observed

All the chickens inoculated with SDSX1 died within 5 days post inoculation and the survival curve was shown in Fig. 3. The median survival of inoculated group was 4 and the statistical differences between groups in survival was obvious significant (*p* < 0.0001). There were no obvious clinical manifestations for the inoculated chickens and the chickens had normal intake before death. Typical gross pathological changes in chickens were the flabby heart with straw-colored fluid in the pericardial sac and the swollen liver with foci of hemorrhages (Fig. 4a). The histopathology revealed that fatty degeneration of hepatocytes, basophilic intranuclear inclusion bodies in hepatocytes and necrosis of hepatocytes (Fig. 4b). All control chickens, the instead of that did not show any symptoms at the end of the observation.

### Virus load analysis

**Fig. 5:**
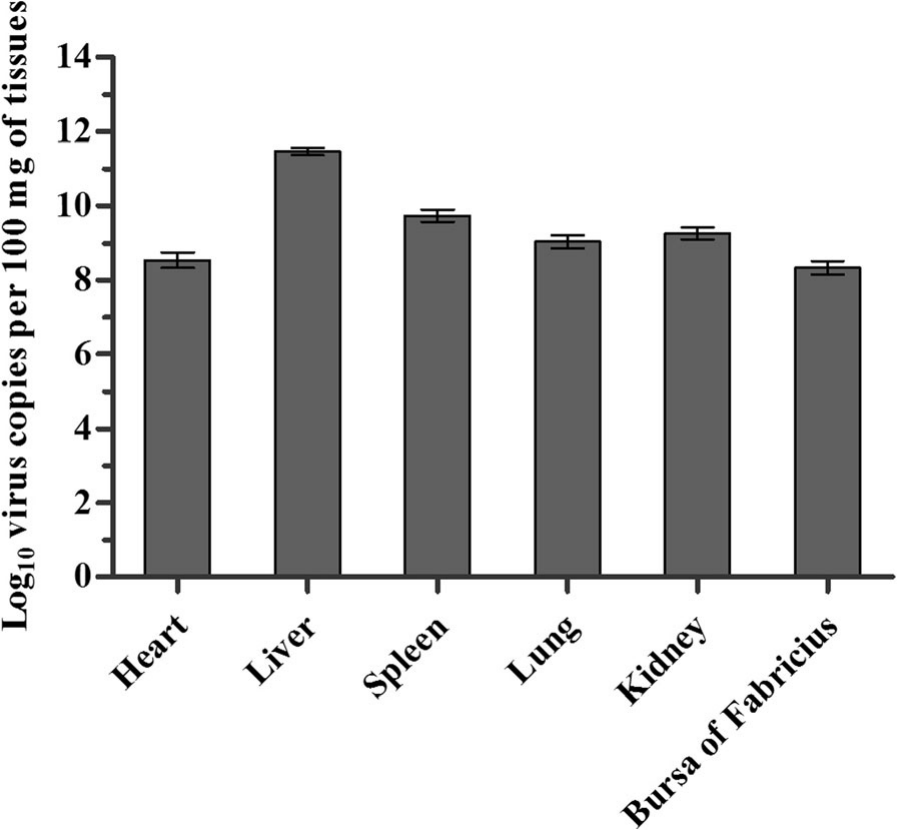
The virus distribution of SDSX1 in experimented SPF chickens

Tissues including heart, liver, spleen, lung, kidney, and bursa of Fabricius were collected and the virus copies in tissues were determined using Real-time PCR. The results showed that virus can be detected in these tissues and the number of virus copies was between 10^7^ and 10^11^ copies per 100 mg tissue with the highest viral genome copies in liver (Fig. 5).

## Discussion

Since 2015, FAdVs infection with high mortality was endemic in the poultry farms in China, resulting in considerable economic losses to the poultry industry [14, 18–21]. It is reported that most cases have been observed in broilers of 3 to 5 weeks of age, and occasionally in layers and breeder pullets aged 10 to 20 weeks and the infection was characterized by hepatitis hydropericardium syndrome (HHS) [10, 22]. In addition to the cases mentioned above, the isolation of FAdV-C from ducks with HHS was also reported [23, 24]. In our study, one strain was isolated from the chickens with HHS infection, and the whole genome was sequenced and analyzed. In addition, pathogenicity of the isolate SDSX1 was evaluated.

Phylogenetic analysis based on the FAdV isolates from different geographic areas worldwide showed that SDSX1 was clustered into serotype-4, FAdV-C species. Nucleotide alignment of SDSX1isolate with theidentity of reference strains in 5 species showed thehigher identity of 98.6–100% with FAdV-C using the complete genome sequence. The alignment of the hexon of SDSX1 had the similar result in the percentage identity compared to the full genome sequence. Especially, SDSX1 had 100% identity with the three isolates in China (CH/SDDZ/2015, CH/AHBZ/2015 and CH/JSXZ/2015).

The data indicated that SDSX1 was a highly pathogenic isolate. Regarding the pathogenicity of the SDSX1 isolate, the survival curve showed that all of 10 one-day-old SPF chickens inoculated with the SDSX1 died within 14 days. The results were consistent with several previous studies in which parts of serotype 4 were highly pathogenic strains [19, 25, 26]. The 100% of mortalityshowed in our study indicated that SDSX1 isolate was a virulent strain, and these were also accounted for the pathogenicity of the virus, serotypes, and dosage of infected strain, especially the susceptibility of the chickens. Different pathogenicity, dynamic distribution and replication pattern in chickens and ducks were observed in the recent report [25].

It is reported that fiber-2 played a significant role in virulence of FAdV [27]. Several scientists’ studies showed that some amino acid residues, positions located at 219 and 380 in the fiber-2 protein of KR5, were related to the virulence based on the amino acid residues comparison between strains induced HHS and non-virulent strains [20, 28]. The fiber-2 protein not only plays a critical role in mediating infection by FAdV-4 but also has a potential capacity to be the candidate vaccine. Immunogenicity of the recombinant fiber-2 protein was evaluated and the results showed that both the level of IgY and the CD4^+^ T cell proliferative response of the chickens immunized with fiber-2 protein were significantly higher than that of the chickens immunized with inactivated vaccine, and fiber-2 could provide 100% protection [29]. The neutralizing epitope recognized by a mAb 3C2 was identified to be located between aa 416–448 in the fiber-2 protein of FAdV-4 and the fiber-2 protein could efficiently block infection by FAdV-4 [30]. In our study, there were more than 20 amino acids residues compared with non-virulent strains (ON1 and KR5), including the position 219(Glycine-Aspartic acid) and position 380(Alanine-Threonine) which had been identified. Whether the remaining amino acid resides substitutions of fiber-2 is responsible for the virulence of SDSX1 is unknown and need to be further studied in future.

## Conclusions

In conclusion, the isolate SDSX1, identified as FAdV-4, could cause in one-day-old chicks’ typical inclusion body hepatitis (IBH) and hepatitis-hydropericardium syndrome (HHS) with 100% mortality. The virus genome loads were highest in the liver. Molecular analysis indicated that substitutions in fiber-2 proteins may contribute to the pathogenicity of SDSX1.

## Methods

### Samples used in this study

Twenty 10~20-day-olddead “817” broilers were collected from various farms in Liaocheng in Shandong province. Gross lesions were examined, and liver tissues were fixed in 10% formalin for 48 h at room temperature and then HE staining was performed following routine histological procedures. Animal work performed in this study was approved by Research Animal Use and Care Committee, the Agricultural University of Hebei, in compliance with the Guidance for the Care and Use of Laboratory Animals issued by the Ministry of Science and Technology of China.

### Viral DNA extraction and PCR

A TaqMan-based real-time PCR assay developed in-house for specific detection of FAdV-4 was used to determine the presence of FAdV-4 in collected liver samples as described previously [31]. The primers sequences were: sense, 5’-TTACGCTTACGGTGCCTACGT-3′;and antisense, 5’-CCGCGTTATTCATGATCCAGTA-3′. The TaqMan probe was as follows, FAM-5’CGACGGTTCCCAGTCCCTCACG-3′-Eclipse. The product size was 89 bp. Briefly, 10 g liver tissue was homogenized in 5 ml 0.01 M PBS. After freeze thawing for three times, the samples were centrifuged in 10000 rpm for 10 min. The supernatants of each sample were collected for viral DNA extraction. Viral DNA extraction was done TIANamp Virus DNA kit (Tiangen Biotech Co., Ltd., Beijing, China) according to the manufacturer’s instructions. The viral DNA from each sample was eluted in a final volume of 100 μl of nuclease-free water.

### Next generation sequencing

The whole sequence of the isolated virus was sequenced by Next Generation Sequencing (NGS). Briefly, the DNA samples were ultrasonicated to generate fragments less than 500 bp. The DNA fragments were polished using T4 polynucleotide kinase. The adaptors were then ligated and loaded on the HiSeq 2000 for sequencing.

### Virus isolation

Virus isolation was performed on the samples with positive PCR results. The liver tissue was homogenized in 0.01 M PBS containing penicillin (100 IU/μL) and streptomycin (100 μg/μL) with the ration of 1:5(*W*/*V*), filtered and centrifuged. The supernatant from each sample was then collected and used for inoculation of 9-day-old specific pathogen free (SPF) chicken embryos (Beijing Vital River Laboratory Animal Technology Co., Ltd.) via allantoic cavity. The dose of inoculation was 0.1 ml per embryo. The allantoic fluid from infected embryos was harvested and used for repeated inoculation of chicken embryos. 5 embryos were used for each inoculum. The inoculation dose of allantoic fluid was 0.1 ml per embryo for each time. After 5 passages, allantoic fluid was harvested in the biohazard safety equipment and virus nucleic acid was extracted for DNA sequencing analysis. A specific fragment of 724 bp from the hexon gene was amplified by PCR for detecting the virus and the following primers were used: sense, 5’GACCTTCGCGGACTACTTGG3’; and antisense, 5’GACGGTTTGGTTGGAACTGAT3’.

### Sequence analysis

To compare the complete genomic sequence and partial hexon gene sequence of the isolate determined in this study, 11 published genomic sequences available in GenBank (Table 1) were aligned by the MegAlign program of the DNAStar software version 7.0 with the Clustal W method (DNASTAR, Madison, Wisconsin, USA). The phylogenetic tree was constructed using the Maximum-Likelihood (ML) approach and MEGA6.06 [32] on the aligned data set. The analysis of amino acids residues of fiber-2 of SDSX1 isolate was done using DNAStar software version 7.0.

### Determination of EID_50_

Virus titer in chicken embryos for the allantoic fluid of five passage viruses was determined by chicken embryos infectivity assay. The result was recorded as EID_50_ per milliliter by using the Reed-Muench method. Briefly, 9-day-old chicken embryos were inoculated the allantoic fluid of the fifth passage viruses (0.1 ml/embryo), which were prepared by serial 10-fold dilution. The embryos were incubated at 37 °C for 6 days and observed every day. Embryos that died within the first 24 h were discarded, those that died than 24 h were selected for the allantoic fluid collection. PCR was used for detecting virus in allantoic fluid. Results were recorded.

### Pathogenicity analysis of SDSX1 isolate

Twenty one-day SPF chickens were randomly allotted to two groups (10 chickens per group). For the inoculated group, each chicken was intraperitoneally inoculated with 1 ml SDSX1 virus containing 10^6^ EID_50_ per chicken. Virus used for inoculation was prepared in the biohazard safety equipment. Chickens in the control group were mock inoculated with the same dosage of PBS. The dosage and pathway of inoculation was selected based on the pre-experiment (data not shown). All the chickens were observed daily for clinical signs up to all chickens are dead. The date and time of the death of each animal were recorded. When the chickens died, the liver samples were collected for the histopathological examination and virus detection.

### Virus load analysis

Quantification of the SDSX1 load in each of the analyzed organs was performed by Real-time PCR method reported in the previous study [31]. Briefly, an equal amount of heart, liver, spleen, lung, lung, kidney and bursa of Fabricicus (100 mg each) from each bird was homogenized in 2 ml PBS. Viral DNA extraction was done according to the method mentioned above. The viral DNA from each sample was eluted in a final volume of 100 μl of nuclease-free water. The TaqMan real-time PCR was performed in a final volume of 25 μl containing 2 × premix Ex Taq Master Mix12.5 μl, 1 μl of 10 μM primers, 0.8 μl of 10 μM probes, 1 μl DNA template and 8.7 μl RNase-free water. The reaction was carried out in ABI 7500 Real-Time PCR System and the optimal reaction procedure was as follows: pre-denaturing for 30s at 95 °C, followed by 40 cycles of denaturation at 95 °C for 10s, annealing at 65 °C for 35 s. A standard curve was used to calculate the virus copies: Y = − 3.56LogX + 46.08. “Y” indicates cycles and “X” indicates copy number.

### Statistical analysis

Data were expressed as mean ± standard deviations. The survival curve of the inoculated and control chickens and the photograph of SDSX1 virus distribution in experimented SPF chickens were made using GraphPad Prism (version 5.0). One-way analysis of variance test was used.

## Abbreviations

bp: Base pairs
EID_50_: Egg 50% infectious dose
HE: Hematoxylin &Eosin;
PBS: Phosphate buffer solution
PCR: Polymerase chain reaction
